# Short-Term Effects of Harassment, Racial Mistreatment, and Incivility (HARM) on Career-Derailing Attitudes: An Experience Sampling Methodology Study

**DOI:** 10.3390/bs16020214

**Published:** 2026-02-02

**Authors:** Jessica M. Kiebler, Amanda E. Mosier, Wei Wu, Ann C. Kimble-Hill, Margaret S. Stockdale

**Affiliations:** 1 Department of Psychology, Indiana University Indianapolis, Indianapolis, IN 46202, USA; 2 Zurich North America, Schaumburg, IL 60196, USA; 3 Department of Biochemistry & Molecular Biology, Indiana University School of Medicine, Indianapolis, IN 46202, USA

**Keywords:** harassment, microaggressions, incivility (HARM), experience sampling methodology (ESM), attitudes, race

## Abstract

Past research has consistently demonstrated the negative effects of interpersonal mistreatment on student experiences by employing retrospective studies; however, little is known about the daily effects that could lead to career derailment. The present study advances evidence of the consequences of experiencing multiple forms of interpersonal mistreatment, including sexual harassment, racial harassment and microaggressions, and incivility (collectively labeled HARM) by employing an experience sampling methodology (ESM) to estimate the immediate impact of HARM on career-relevant attitudes among a sample of 202 biomedical health trainees (mentees) funded by a National Institutes of Health fellowship. Grounded in Affective Events Theory, we found that mentees’ daily experiences of HARM were associated with an immediate degradation of their attitudes toward their training program mediated by negative affect. Being racially isolated in a lab or having a racially different mentor increased the prevalence of HARM; moreover, accounting for negative affect, experiences of HARM were positively associated with program attitudes for mentees who were racially well-represented, suggesting that majority status may buffer the negative impact of HARM on attitudes. Understanding these dynamics provides insight into the importance of assessing and addressing daily experiences of mistreatment among graduate and postdoctoral trainees.

## 1. Introduction

Graduate and postdoctoral research training programs serve as a critical gateway to careers in biomedical science, offering specialized expertise, enhanced employment opportunities, and access to professional networks that shape long-term career trajectories. Individuals with advanced degrees experience greater lifetime earnings, higher job stability, and increased opportunities to influence scientific and societal outcomes (Day & Newburger, 2002; Irwin et al., 2022). Reflecting on these benefits, enrollment in biomedical graduate programs has expanded substantially over the last four decades, including increased representation of women and students from historically underrepresented racial and ethnic groups (NIH, 2025). These demographic shifts represent important strides toward diversifying the biomedical workforce and broadening perspectives in scientific discovery.

However, the pursuit of advanced training is not without substantial psychological and interpersonal costs. Graduate students and postdoctoral fellows frequently report elevated stress, emotional exhaustion, and significant rates of interpersonal mistreatment within training environments (Cho & Hayter, 2020; Kurd & Hummel, 2023; Lorenz et al., 2019). Mistreatment—including sexual harassment, racial harassment and microaggressions, and interpersonal incivility (collectively referred to as HARM)—has been documented in approximately one-third to one-half of trainees, with disproportionately higher incidence among women and Students of Color (Buchanan et al., 2009; Goodboy & Martin, 2015; Rosenthal et al., 2016).

Sexual harassment is a set of unwanted sex- or gender-based behaviors—including sexual coercion, unwanted sexual attention, and gender harassment—that function to insult, intimidate, exclude, or subordinate individuals on the basis of sex with the goal of reinforcing patriarchal norms of gender hierarchy (Cortina & Areguin, 2021; Fitzgerald et al., 1997; Stockdale & Stalion, 2025). Racial harassment consists of overt or subtle behaviors that demean, intimidate, or exclude individuals on the basis of race, communicating hostility toward their racial identity and undermining their psychological safety and belonging (Buchanan et al., 2009; Daniel et al., 2023). Racial microaggressions are brief, commonplace verbal, behavioral, or environmental slights that convey derogatory, dismissive, or negative messages to people of color, often unintentionally, and cumulatively erode well-being and sense of belonging (Sue et al., 2007). Last, but often experienced most frequently, is incivility which includes low-intensity deviant behavior that violates norms of respect—such as rudeness, dismissiveness, or condescension—while carrying ambiguous intent to harm but nonetheless impairing psychological well-being and work engagement (Andersson & Pearson, 1999; Cortina et al., 2013; Namin et al., 2022). Such mistreatment undermines academic engagement, disrupts research productivity, diminishes confidence, and contributes to attrition from STEM fields, threatening efforts to build a diverse and sustainable scientific workforce.

A large body of research demonstrates that the components of HARM—sexual harassment, racial harassment, racial microaggressions, and incivility—frequently co-occur and operate through shared psychological mechanisms, justifying their integration into a unified construct of interpersonal mistreatment. Cortina and colleagues work on selective incivility shows that ostensibly “neutral” disrespect often reflects underlying systems of sexism and racism, meaning that identity-based and non-identity-based mistreatment emerge from the same discriminatory climates (Cortina, 2008; Cortina et al., 2013). Similarly, Hershcovis’ meta-analytic research reveals that different forms of workplace aggression produce highly similar emotional, cognitive, and attitudinal outcomes, suggesting that distinctions between constructs are often more semantic than substantive (Hershcovis, 2011; Hershcovis & Reich, 2013). Empirical studies consistently demonstrate overlapping antecedents, correlated experiences, and convergent outcomes—including heightened negative affect, reduced satisfaction, increased psychological distress, and withdrawal (Berdahl & Moore, 2006; Buchanan et al., 2009; Lim et al., 2008; Junça-Silva et al., 2021). Furthermore, as discussed below, Affective Events Theory (AET; Weiss & Cropanzano, 1996) further supports a collective approach by arguing that it is the affective impact of negative interpersonal events—rather than their specific categorical labels—that drives changes in attitudes and behavior. Thus, studying these experiences together as HARM provides a theoretically coherent and empirically grounded framework for capturing the cumulative and affective burden of mistreatment in academic training environments.

Despite compelling evidence that HARM exerts negative consequences on long-term academic and career outcomes, there is limited empirical work examining the immediate psychological and attitudinal effects of daily mistreatment in research training environments. Immediate consequences matter because they signal the beginning of cognitive and affective processes that may accumulate over time and contribute to disengagement, performance declines, and eventual withdrawal from training. To understand these short-term processes, researchers require theoretical and methodological frameworks that capture dynamic, within-person responses to daily experiences.

Affective Events Theory (AET) offers a framework for explaining how daily interpersonal experiences—particularly negative interpersonal events—shape momentary affective states and subsequently influence attitudes and behaviors (Weiss & Cropanzano, 1996). Integrating AET into the study of HARM in academic training environments provides insight into why even a single incident of mistreatment can meaningfully influence students’ satisfaction, motivation, and perceived progress toward their degree. By conceptualizing HARM as a negative affective event, and negative affect as the proximal mechanism shaping attitudes toward one’s program (e.g., graduate or postdoctoral training), AET provides a theoretically coherent account of daily fluctuations in trainees’ psychological experience.

The present study applies AET to examine how daily experiences and observations of HARM influence graduate trainees’ affective reactions and, in turn, their attitudes toward their training programs. Furthermore, because identity-related factors shape the meaning and emotional impact of interpersonal events, we incorporate tokenism and relational demography to examine whether gender and race composition—both at the lab level and within mentoring dyads—moderate these AET pathways.

### 1.1. Literature Review

To date, research on harassment, racial mistreatment, and incivility (i.e., HARM) in academic and organizational contexts has documented extensive long-term associations between mistreatment and detrimental outcomes, including decreased satisfaction, impaired performance, impaired psychological health, and withdrawal from academic or professional environments. Although this body of research is foundational, a key limitation is that much of it relies on cross-sectional or retrospective evaluations that cannot fully capture the immediate psychological mechanisms through which negative interpersonal events shape attitudes and behavior. Affective Events Theory (AET; Weiss & Cropanzano, 1996) provides a theoretically robust lens through which to understand these proximal processes.

#### 1.1.1. Affective Events Theory as a Framework for Understanding HARM

AET asserts that workplace events—especially interpersonal events—serve as triggers for short-term emotional reactions that subsequently shape work attitudes (e.g., job satisfaction, commitment) and behavior, such as withdrawal. Negative interpersonal encounters such as incivility, harassment, or microaggressions constitute “affective events” because they are salient, socially evaluative, and capable of evoking immediate emotional states such as anger, anxiety, fear, or humiliation (Ashton-James & Ashkanasy, 2005). These emotional responses, rather than the events themselves, are posited to be the proximal predictors of downstream evaluative judgments, and empirical research consistently shows that negative interpersonal experiences reliably elicit increases in state negative affect, which in turn predict lower satisfaction, commitment, and engagement (Lim et al., 2008; Junça-Silva et al., 2021; Weiss & Cropanzano, 1996). Thus, the model predicts a mediation pathway in which HARM predicts negative affect, which in turn predicts negative job or training program attitudes.

Contemporary empirical work supports this sequence. As we expand on below, daily diary and experience sampling methodology (ESM) studies consistently show that interpersonal mistreatment elicits negative affective reactions that subsequently forecast decrements in satisfaction, commitment, and well-being (Demerouti & Cropanzano, 2017; Junça-Silva et al., 2021). In studies of microaggressions and racialized mistreatment, momentary experiences of bias have been shown to heighten negative affect and reduce well-being, belongingness, and engagement later the same day (Junça-Silva & Ferreira, 2025). Similarly, daily incivility predicts elevated stress and anger, which in turn predict reduced engagement and increased withdrawal (Lim et al., 2008). Although sexual and racial harassment are often less frequently observed in daily designs, analogous affective pathways have been documented in cross-sectional and longitudinal work showing that emotional reactions and stress serve as central mediators between harassment and job attitudes (Fitzgerald et al., 1997; Buchanan et al., 2009).

Collectively, this research positions mistreatment as a potent generator of affective disruption that meaningfully shapes individuals’ attitudes toward their work or academic settings. As graduate students’ learning environments are characterized by power-dependent interpersonal relationships, high demands, and tightly coupled social interactions, AET suggests that even a single negative interpersonal event may produce a perceptible shift in affect and engagement.

#### 1.1.2. Daily Diary and Experience Sampling Evidence for the AET Mediation Model

AET further proposes that affective reactions unfold in real time and vary meaningfully within persons across days. Thus, methods capable of capturing intraindividual variability, such as ESM, are particularly well-suited for testing the temporally proximal pathways proposed by the theory. Research using ESM has demonstrated that daily workplace events—both positive and negative—predict fluctuations in affect that subsequently predict same-day or next-day satisfaction, performance perceptions, and social behavior (Ashton-James & Ashkanasy, 2005; Demerouti & Cropanzano, 2017)

The methodological advantages of ESM map directly onto AET propositions. First, ESM minimizes retrospective bias, allowing for more precise measurement of ephemeral affective states. Second, ESM permits disentangling within-person processes, aligning with AET’s claim that affective reactions fluctuate daily and should be modeled at the event level. Third, ESM enables time-ordered tests of mediation, allowing researchers to observe whether negative events precede affective shifts and whether these shifts precede changes in attitudes. In short, ESM represents the optimal methodological approach to evaluate the microprocesses underlying AET.

#### 1.1.3. HARM as a Negative Interpersonal Affective Event in Academic Contexts

Harassment, racial microaggressions, and incivility represent quintessential negative affective events in AET because they violate norms of respect, signal devaluation, threaten identity, and undermine expectations of interpersonal safety. Such events are especially consequential in academic research settings where trainees rely heavily on mentors, peers, and lab groups for social belonging, evaluation, sponsorship, and career progression (Johnson-Bailey & Cervero, 2007; Ragins, 1997). In these settings, mistreatment is not simply an interpersonal slight—it is a threat to academic identity, career trajectory, and psychological safety.

Recent studies applying AET to incivility and microaggressions confirm that mistreatment predicts daily negative affect, which subsequently predicts poorer well-being, reduced engagement, and more counterproductive behavior (Junça-Silva et al., 2021; Junça-Silva & Ferreira, 2025). These findings align with research showing that racially biased interactions can undermine belonging and heighten vigilance, producing immediate affective costs (Civitillo et al., 2024). Theoretical work on ambient harassment and microaggressions similarly suggests that the psychological harm of mistreatment occurs at the level of daily emotional disruption (Glomb et al., 1997; Nadal et al., 2014). Applying these insights to graduate education suggests that daily HARM may produce immediate affective consequences that cascade into negative program attitudes.

#### 1.1.4. Identity-Based Moderators: Tokenism, Relational Demography, and AET

AET acknowledges that individuals differ in how strongly they react to affective events, and recent theoretical expansions emphasize that identity-based factors influence event appraisal and affective reactivity. Two categories of moderators are particularly relevant: tokenism and relational demography.

Tokenism theory (Kanter, 1977) posits that individuals who are numerically underrepresented in their environment experience greater scrutiny, identity threat, and social isolation. These contextual pressures heighten sensitivity to negative interpersonal events, making mistreatment more salient and more emotionally taxing. Empirical evidence supports these mechanisms: being the sole or one of few women or racial minorities in a group increases the likelihood of experiencing harassment and intensifies its negative psychological effects (Berdahl & Moore, 2006; Sutton et al., 2021). AET predicts that tokens will exhibit stronger affective responses to mistreatment because events violating belongingness or identity norms carry greater diagnostic meaning (Purdie-Vaughns et al., 2008).

Research on relational demography and identity safety suggests that demographic mismatch between leaders and followers can heighten vigilance for bias and intensify affective reactions to negative interpersonal events. Foundational work by Tsui and O’Reilly (1989) demonstrates that demographic dissimilarity in supervisor–subordinate dyads predicts poorer relationship quality, lower perceived support, and more negative evaluations, indicating that identity mismatch shapes interpretations of interpersonal behavior. Identity-safety research further shows that individuals from stigmatized groups are acutely attuned to contextual and interpersonal cues that signal whether “people like me” are valued or devalued (Purdie-Vaughns et al., 2008). Studies by Johnson and Pietri and colleagues reveal that demographically matched role models and leaders serve as identity-safety cues that enhance trust and belonging, whereas mismatched leaders may evoke concerns about bias and heighten the likelihood that ambiguous or negative events are interpreted as identity-relevant (e.g., Johnson et al., 2019; Pietri et al., 2019). Related work demonstrates that the absence of identity-safety cues in STEM and educational contexts increases social identity threat and negative affective responses to interpersonal slights (Emerson & Murphy, 2014). Together, this literature supports the expectation that negative interpersonal treatment (HARM) will be experienced as more threatening—and evoke stronger negative affect—when mentees are paired with demographically dissimilar leaders, because mistreatment is more readily interpreted as reflecting group-based devaluation.

Together, these theoretical perspectives suggest that numeric representation and identity match should moderate the strength of the HARM → affect → attitudes paths hypothesized below.

### 1.2. Hypotheses

Drawing on AET and empirical research on harassment, microaggressions, and incivility, we propose the following: First, HARM represents negative interpersonal events that elicit immediate emotional disruption.

**Hypothesis** **1.** *Daily HARM will be positively associated with daily negative affect*.

Second, consistent with AET, affective reactions are proximal predictors of satisfaction, engagement, and confidence.

**Hypothesis** **2.** *Daily negative affect will be negatively associated with daily program attitudes.*

Third, HARM should indirectly reduce program attitudes through increases in negative affect.

**Hypothesis** **3.** *Daily negative affect will mediate the relationship between daily HARM and daily program attitudes.*

Next, we propose moderators of these effects based on tokenism theory and relational demography theory. First, individuals who are numerically underrepresented will experience stronger emotional reactivity to mistreatment.

**Hypothesis** **4.** *Numeric underrepresentation in (a) lab gender and (b) racial composition will strengthen the positive relationship between daily HARM and daily negative affect*.

**Hypothesis** **5.** *Numeric underrepresentation in (a) lab gender and (b) racial composition will strengthen the negative indirect effect of HARM on program attitudes through negative affect.*

Second, when salient social identities between trainees and their mentors (i.e., gender and race) are not matched, the effects of HARM on negative affect and on program attitudes indirectly through negative effect will be exacerbated.

**Hypothesis** **6.** *Mentor–mentee (a) gender mismatch and (b) race mismatch will strengthen the positive relationship between daily HARM and negative affect.*

**Hypothesis** **7.** *Mentor–mentee (a) gender mismatch and (b) race mismatch will strengthen the negative indirect effect of HARM on program attitudes through negative affect.*

Third, we leave open the possibility that tokenism and relational demography effects will directly moderate the associations between experiences of HARM and program attitudes for two reasons. One reason is that there may be other, unmeasured affective mediators beside the negative affect that could be explaining the effects of HARM experiences on attitudinal outcomes for which tokenism and relational demography constellations could be moderating. A second reason is that in controlling for the negative affect, there may be non-emotional reasons why HARM experiences may impact program attitudes.

**Hypothesis** **8.** *(a) Lab gender, (b) lab race, (c) mentor–mentee gender mismatch, and (d) mentor–mentee race mismatch will moderate the direct (non-affective) association between HARM and program attitudes.*

**Figure 1:**
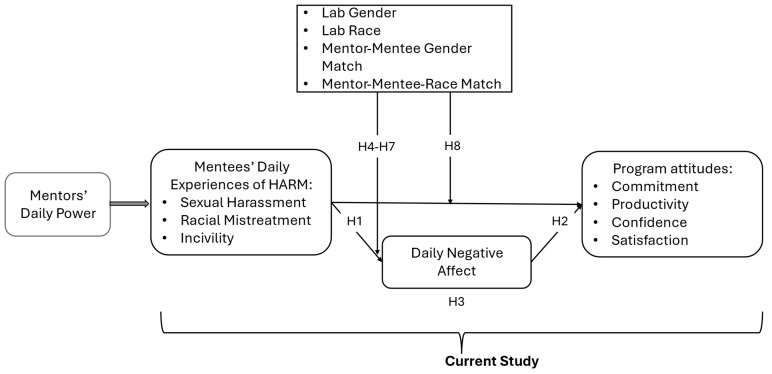
Study design and hypothesized relationships.

Figure 1 depicts the study design and these hypotheses.

## 2. Methods

Student research mentees involved in National Institutes of Health (NIH) funded research were invited to participate in a series of online surveys for 10 days (experience sampling methodology) to assess daily experiences of harassment, racial mistreatment, and incivility (HARM) and their potential effects on students’ attitudes about their educational programs of study while also attending important contextual factors that might influence this relationship (e.g., the racial and gender makeup of their lab and between the mentors and mentees). The current study was part of a larger study of the effects of research mentors’ daily embodiment of power and feelings associated with power (sexy–powerful feelings, communal feelings, and moral licensing) on one of their mentees’ daily experiences of harassment, racial mistreatment, and incivility (HARM). The current study focuses on the relationship between mentees’ HARM experiences and their program attitudes. Because their HARM experiences emanated from their mentors’ powerful states, we describe the mentor sample here, but we do not report the measures completed by the mentors nor results based on those measures. The manuscript reporting on those measures is under preparation.

### 2.1. Participants

In the current study, the primary goal was to assess daily experiences of HARM among student mentees in lab settings where research was actively being conducted. To systematically recruit researchers currently participating in funded research, the NIH Reporter database of projects provided ample opportunity to find primary investigators on projects where they often mentor students. Researchers funded by the National Institute of Health (NIH) who were principal investigators (PIs) of research focused grants (activity types R01, R21, or R35) were the target population for the mentor sample. One to two of their graduate students or post-doctoral fellows were the target population for the mentee sample. The current study consisted of 202 mentor and mentee dyads.

We first considered a power analysis to determine the number of dyads necessary to test our hypotheses. Gabriel et al. (2019) reviewed the empirical literature on ESM studies in organizational contexts and found little detail on how previous researchers assessed the power of such designs. Therefore, they summarized the average sample sizes for both between-subjects and within-subjects observations, which they reported to be 83 and 835, respectively. Because our study assessed the cross-over effects of mentors’ psychological states on mentees’ experiences of potentially rare events, we expected to find very subtle effects. Therefore, we aimed to recruit as large a sample as possible within the constraints of our resources and sampling frame.

#### 2.1.1. Mentors

We recruited NIH-funded faculty researchers with an active research grant by randomly sampling principal investigators (PIs) from NIH’s Reporter database across three cycles in Spring 2023, Fall 2023, and Spring 2024. Altogether, we sampled 9000 PIs proportionally across the three activity types from 27 Institutes, excluding those who were not affiliated with a college or university. Of these, 359 PIs (mentors) completed an enrollment survey, which asked them to list, among other things, the mentees in their lab along with their demographic information. Those who listed fewer than two mentees were excluded (N = 19).

#### 2.1.2. Mentees

From the list of mentees provided by mentors, we invited two mentees per PI to participate in the study. If we had more than two to choose from, we randomly selected two from their pool of mentees, oversampling women and underrepresented minorities (those other than White or Asian race/ethnicities) to ensure we had large enough sample sizes to look at group differences on mentees that matched and did not match the gender/racial identity of their mentors. Of these, one was randomly selected to serve as the primary mentee for the study. Our final sample consisted of 202 mentor/mentee dyads. Again, for the current study, only the mentee data were analyzed. N’s ranged from 79 to 179 participants per day with a total of 1667 within-person observations across 10 days.

Primary mentees (and mentors) were compensated with a USD 150 Amazon gift card if they completed at least 70% of the daily surveys. In the primary mentee sample, there were 142 (70.3%) who identified as female, 56 (27.7%) who identified as male, and 4 (2.0%) who identified as gender non-conforming, preferred not to self-describe their gender, or did not report their gender. There were 34 (16.8%) who identified as Hispanic or Latinx, any race. Of those who identified as non-Hispanic, 54 (26.7%) identified as Asian or Asian American, 15 (7.4%) identified as Black, 86 (42.6%) identified as White, 5 (2.5%) identified with more than one race, and 8 (4.0) identified with a race not listed on the survey or who did not report their race or ethnicity. The modal degree sought was a Ph.D. (n = 133, 65.8%), followed by “other” (post-doctoral fellows, n = 46, 22.8%), and M.S. (n = 10, 5.0%).

### 2.2. Measures

Mentees completed measures of lab gender, the extent to which the people working with their primary research mentor are (1) one or very few people of the same gender as them to (5) all or almost all of the same gender as them (the midpoint was “about the same number of people with the same gender as me as well as people with a different gender than me”). *Lab race* was measured with a similar scale replacing gender with “race/ethnicity.”

Similarly, mentees and mentors were given scores on whether or not they matched each other in terms of gender and racial identity. The variables of *mentor/mentee race* (MMrace) and *mentor/mentee gender* (MMgender) were created to capture similarities and differences in identity between mentors and mentees. Scores of zero indicate that they were not matched in terms of gender or racial identity, and scores of one indicated they did match. Mentees were considered a match with their mentor on race if they were both either well-represented majority members (i.e., non-Hispanic White, Asian, or White-Asian) or both underrepresented minority members (i.e., Hispanic or race other than White, Asian, or White Asian), since precise race matching yielded too few matches for underrepresented minority mentees.

Mentee lab gender/race and mentor/mentee gender and race were asked during the intake survey while program attitudes and daily experiences of HARM were asked in each of the 10-day surveys.

#### 2.2.1. Program Attitudes

To assess short-term attitudes that signal persistence versus potential derailment in graduate and postdoctoral training, we focused on four core dimensions that prior research has consistently linked to retention-related outcomes. Attitudes reflecting commitment to one’s organization or program, perceived effectiveness and productivity, confidence in one’s abilities, and overall satisfaction have repeatedly emerged as central predictors of sustained engagement and lower withdrawal intentions across work and training contexts (N. J. Allen & Meyer, 1996; Christian et al., 2011; Griffeth et al., 2000; Halbesleben, 2010; Meyer et al., 2002; Pohlan & Steffes, 2025; Saks, 2006). Meta-analytic evidence indicates that individuals who report stronger commitment, higher satisfaction, greater self-efficacy, and more positive performance perceptions are more likely to remain engaged and less likely to disengage or exit their roles (Christian et al., 2011; Griffeth et al., 2000).

Consistent with this literature, program attitudes were operationalized using four items assessing participants’ momentary evaluations of their training experience over the past 24 h: (a) commitment to remaining in the program, (b) perceived productivity, (c) confidence in one’s abilities, and (d) overall satisfaction with the graduate or postdoctoral program. Responses were recsorded on 5-point scales ranging from 1 (much lower than normal) to 5 (much higher than normal). Scores were averaged to form a composite Program Attitudes index (ProgAtt). These items were averaged to create the scale of *Program Attitudes.* Internal consistency reliabilities (α) ranged from 0.77 to 0.87 across ten days.

#### 2.2.2. Measures of HARM

Measures of HARM were derived from shortened versions of well-established scales of sexual harassment, racial harassment, racial microaggression, and incivility. Answers to each item on the following scales were recorded on a 3-point scale: (0) *Not at all* (1) *Somewhat*, or (2) *Yes, definitely*. Although we estimated the internal consistency (α) of the items in each measure of HARM as well as the HARM composite variable, we caution the interpretation of this statistic because each item measures a specific experience, which may not correlate with another experience on the scale. In other words, the scales measure formative constructs, not reflective constructs (K. A. Bollen & Diamantopoulos, 2017; K. Bollen & Lennox, 1991; cf. Edwards, 2010/2011).

*Sexual Harassment* was measured with a 4-item scale measuring the four forms of sexual harassment commonly assessed (Stark et al., 2002): *Sexist Hostility* (in the past 24 h, someone in my lab engaged in sexist behavior toward me or others”), *Sexual Hostility* (in the past 24 h, someone in my lab engaged in sexually crude behavior toward me or others), *Unwanted Sexual Attention* (in the past 24 h, someone in my lab gave me or others unwanted sexual attention), and *Sexual Coercion* (in the past 24 h someone in my lab implied that I or others would be treated differently if we cooperated sexually with them). We asked about both direct experiences and observations of the experience because prior research has found that both have harmful consequences (Glomb et al., 1997). There were several days when the frequency of sexual harassment experiences was 0.0, which precluded analyses on this outcome separately. On days with a sufficient number of incidents, the reliability of this scale ranged from 0.65 to 0.94.

*Racial mistreatment* was measured with two items adapted from the Sexual Experiences Questionnaire-Latina (Cortina, 2001). Using the same stem as the sexual harassment items, these items were “engaged in racist behavior toward me or others,” and “engaged in racially crude behavior toward me or others. In addition, we added five items adapted from the Racial and Ethnic Microaggression Scale (Nadal, 2011). Using the same stem as above, the items were: “made assumptions that I or other minorities were inferior,” “treated me or other minorities as a second-class citizen,” “invalidated me or other minorities’ experiences as a person of color,” ‘was subtly aggressive toward me or other minorities,” and “ignored me or others minorities or made us feel invisible)”. The frequency of racial mistreatment experiences was very low on several days (less than 5 mentees reporting a value of 1 or higher), which precluded analyses on this outcome separately. The reliability of this scale ranged from 0.82 to 0.94) on days with a sufficient number of incidents on which to assess reliability.

*Incivility* was measured with three items adapted from a scale developed by Lim and Cortina (2005). Using the same stem as above, the items were as follows: “put me down or was condescending to me or others,” “paid little attention to my or others’ opinions,” and “addressed me or others in unprofessional terms either publicly or in private.” Reliabilities ranged from 0.30 to 0.83 (again we caution interpreting internal consistency reliability for formative measures).

*HARM* was a composite measure composed of the sum of sexual harassment, racial harassment, racial microaggression, and incivility. Reliabilities ranged from 0.38 to 0.89.

#### 2.2.3. Negative Affect

*Negative Affect* was measured with the negative affect items from the state Positive and Negative Affect Scale (Watson et al., 1988). Participants rated on scales ranging from (1) *not at all* to (5) *very much* the extent to which they felt currently felt scared, afraid, upset, etc. Reliabilities ranged from 0.80 to 0.86.

### 2.3. Procedure

Ethical approval for this study was granted by the host university’s institutional review board as an exempt protocol, #15142. This study was granted exempt status based on the IRB review that our study posed no more than minimal risk to participants. All participants were provided with the study information sheet (similar to an informed consent statement) which explained study procedures, data protection, confidentiality, the risks and benefits of participation, and compensation for participation. They were also assured that mentors would not see their mentee’s responses, nor would mentees see their mentor’s responses.

After completing the intake survey, which assessed demographics, lab characteristics, and lab tolerance for harm, participants completed ten surveys online via the Qualtrics platform on ten consecutive days starting on a Monday, excluding the weekend. Each participant’s daily survey was yoked to a survey completed by their mentor between 12:00 p.m. and 4:00 p.m. in their time zone. Mentees’ surveys arrived in their inbox at 5:00 p.m. in their time zone and they were asked to complete the surveys by 10:00 p.m.

### 2.4. Data Analysis

Multilevel structural equation modeling (MSEM) with Bayes estimator was used in Mplus 8.9 to test the hypotheses given its flexibility and capability to handle models for all aims. Specifically, it allows us to (a) account for the clustered nature of the data (repeated measures nested within individuals), (b) separate the within- and between-individual effects, (c) automatically handle missing data in model estimation process (Enders et al., 2013), and (d) provide 95% credibility intervals (CIs) that are comparable to bootstrap confidence intervals for significance tests of indirect effects when mediation is involved (Preacher et al., 2010; Preacher et al., 2016; Yuan & MacKinnon, 2009). Note that Bootstrap confidence intervals are not available for multilevel models in Mplus. The analyses were performed only on any HARM since sexual harassment and racial mistreatment scales had very few to no observations on more than one day. Specifically, we first examined the within-individual effect of HARM on program attitude separately (hypothesis 1). We then included lab gender and lab race (hypothesis 2), respectively, as moderators of the effect of HARM. Finally, we included mentor/mentee gender (MMgender) and mentor/mentee race (MMrace), respectively, as moderators of the effect of HARM.

We have checked normality assumption of continuous variables and used trace plots, potential scale reduction value (PSR) < 1.1 criterion, and a large number of (5000) burn-in iterations to ensure that the Bayesian estimator appropriately converged for all models (Asparouhov & Muthén, 2010). To ensure adequacy of the models, we included all possible fixed and random effects. Specifically, the effects of all within-individual predictors (e.g., HARM) were modeled as random (i.e., vary across individuals and have their own distributions). The means and variances for these individual-specific effects were estimated. When there are multiple random effects, these random effects were allowed to be correlated. All the variables were allowed to be freely correlated at the between-individual level. The detailed specification can be found in the MPlus syntaxes provided in the Supplementary Materials referenced at the end of this paper. We obtained both unstandardized and standardized (as effect size measures) estimates for fixed effects. R-squared measures were also reported to indicate the overall predictive power of target predictors. With a possible maximum participation rate of 202 participants, Table 1 reports the daily N’s for reports of each variable. As stated above, MSEM accounted for missing values.

## 3. Results

### 3.1. Descriptive Statistics

The descriptive statistics of the model variables are presented in Table 2. For within-person level variables, as they have daily measures over 10 days, we reported the average prevalence and range of prevalences of those reporting a score of 1 or greater on HARM, and we report the grand means and standard deviations of each of the daily measures as well as the range of means and standard deviations of the daily measures across 10 days. We also reported their intraclass correlations (ICCs). As shown in Table 2, ICCs ranged from 0.29 to 0.55, indicating substantial clustering (Chen & Chen, 2021), which warrants multilevel modeling. The correlation matrices at the within-dyad and between-dyad levels are shown in Table 3 and Table 4, respectively. Also, while HARM was not significantly correlated with program attitudes; negative affect is significantly correlated in expected directions with both measures of HARM and with program attitudes as outlined in hypotheses 1 and 2.

For sexual harassment, average prevalence of experience among those who filled out a survey on a given day was 1.19% (10% to 61% missing data). For racial microaggressions, the average prevalence rate was 3.93% (10% to 60% missing data). Finally, for incivility, the average prevalence was 7.02% (10% to 60% missing data).

Although not the focus of the current study, we note that there were negative correlations between HARM and MMRace and between HARM and Lab race (see Table 4), indicating that HARM experiences were more prevalent among mentees who did not share the same race identity as their mentor or who were in labs with few, if any, other people who shared the same race as them.

### 3.2. Effects of HARM on Program Attitudes

At the within-person level, the average of the individual effects of HARM on program attitudes was not significant; *B* = −0.09, *β* = −0.034, *p* = 0.18. Note that *B* and *β* represent unstandardized and standardized coefficients, respectively. However, significant variance in the effect was detected, *B* = 0.06, *p* < 0.001, indicating substantial individual variability of the effect. Harm accounted for 1.5% (*p* < 0.001) within-level variance of program attitude.

### 3.3. Mediation Analysis

**Figure 2:**
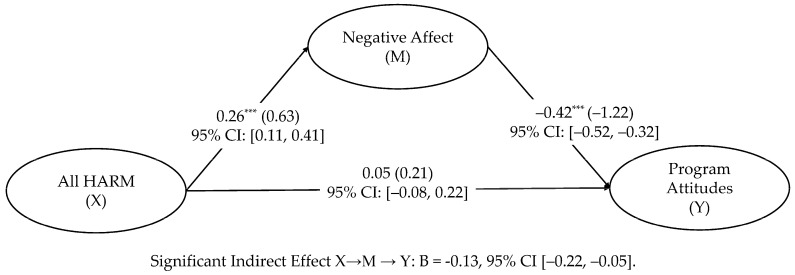
Mediated effects of HARM on program attitudes through negative affect. *** *p* < 0.001. Note. Unstandardized coefficients are outside the parentheses, and standardized coefficients are in the parentheses.

Supporting our third hypothesis, the mediation analysis found that HARM increased negative affect, *B* = 0.26, *β* = 0.63, *p* < 0.001, which in turn decreased program attitudes, *B* = −0.42, *β* = −1.22, *p* < 0.001. The indirect effect was −0.13 with 95% CI [−0.22, −0.05]. At the within-individual level, the model accounted for 16.3% variance in program attitude and 8.9% of the variance in negative affect. These findings are depicted in Figure 2.

### 3.4. Moderation Analysis

**Figure 3:**
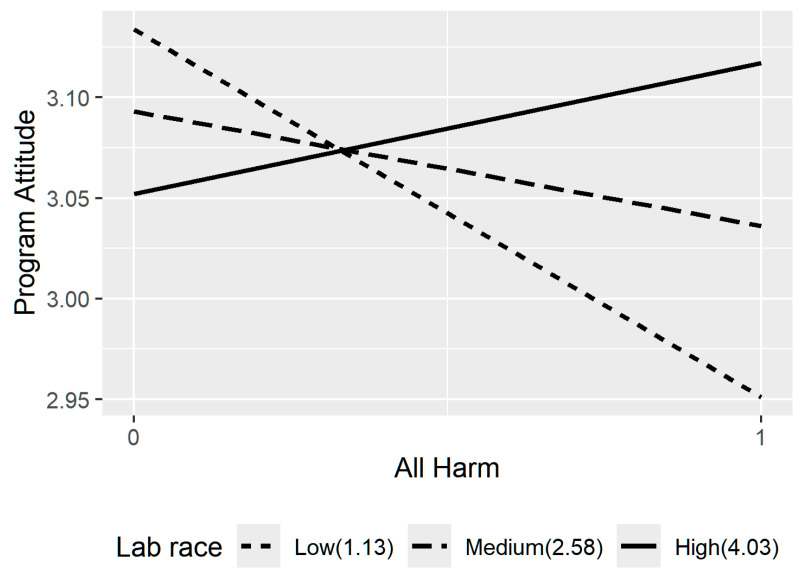
Mistreatment effects on program attitudes moderated by lab race.

We also tested whether lab gender, lab race, MMgender, or MMrace moderated the path between HARM on negative affect; the direct path between HARM on program attitudes, and the indirect effect through negative affect. The moderation analysis results are shown in Table 5 and indicated that only lab race moderated the direct effect of HARM on program attitudes after accounting for the indirect effect via negative affect, *B* = 0.08, *β* = 0.40, *p* = 0.02 (supporting hypothesis 8(b)). Examining the direct HARM effect at three levels of lab race (low: mean − 1SD, moderate: mean, high: mean + 1SD) suggested that the direct effect of HARM became more positive as lab race increased (meaning mentees who were in lab settings where they shared a racial identity with many others). It was significantly positive, *B* = 0.19, *p* = 0.05, when lab race was high. The effect of HARM was not significant under either low *B* = −0.05, *p* = 0.63, or moderate lab race *B* = 0.07, *p* = 0.29. In addition, lab race explained about 16% variance in the direct effect of HARM. See Figure 3 for details.

Hence, while we found that negative affect mediated the effect of HARM experiences on program attitudes, lab race did not moderate this effect—it only moderated the direct effects, therefore, discounting hypothesis 4 and 8b, but in an unexpected direction (e.g., when students were in lab settings and shared a similar racial identity with others), experiences of HARM predicted more positive program attitudes compared to if they were in a lab setting with few other people who shared their racial identity.

## 4. Discussion

The present study contributes to the growing literature on workplace harassment and mistreatment by examining the daily, short-term effects of harassment, incivility, and racial microaggressions (HARM) on biomedical mentees’ attitudes toward their academic programs. Using experience sampling methodology (ESM) to account for daily and immediate effects of HARM, we found that daily experiences of HARM were indirectly associated with more negative program attitudes through elevated state negative affect. Supporting our theoretical basis of AET, these findings underscore the central role of emotional processes in explaining how mistreatment shapes trainees’ perceptions of their academic environments, even over short time intervals. Specifically, while we find no direct effect of experiencing HARM on negative program attitudes among mentees, we do find this relationship to be significant when considering the role of affective emotional responses to experiences of mistreatment. This supports the literature on AET that demonstrates how events themselves are generally not what underlies negative downstream consequences but rather the negative affective experiences of those events (Ashton-James & Ashkanasy, 2005; Lim et al., 2008; Junça-Silva et al., 2021; Weiss & Cropanzano, 1996). Further, supporting previous research related to individual experiences of mistreatment, our findings highlight the importance of diverse methodology in assessing the immediate and culminating effects of mistreatment in the literature on sexual harassment, racial microaggressions, and incivility.

Additionally, contextual analyses suggest that racial representation within laboratory environments moderated the HARM–attitude relationship. Although the extent to which one was numerically minoritized in the lab (i.e., low lab race rating; having a mentor with a different racial identity) was correlated with higher incidents of HARM and predicted more negative program attitudes, we found that HARM positively predicted program attitudes for mentees who shared racial identities (in terms of URM status) with others in their labs. The reversal of the negative effect of HARM on program attitudes points to a suppressor effect, whereby negative affect statistically masks a more positive (non-affective) component of experiencing mistreatment in these contexts specifically (Krus & Wilkinson, 1986; MacKinnon et al., 2000; Rucker et al., 2011).

When the effect of HARM is assessed while parsing out affective distress associated with these experiences, the remaining variance in mistreatment appears to be associated with greater program engagement or collective awareness in racially supportive contexts. Affective Events Theory supports this finding that negative experiences, such as sexual harassment and incivility have a proximal effect on negative feelings (negative affect) that produce more negative attitudes toward the context in which the events are occurring (lower program attitudes) (Weiss & Cropanzano, 1996). However, once those negative feelings are accounted for, in racially homogenous groups, we see the negative contextual feelings reverse pointing to an area for future research to investigate other potential variables influencing this inverse relationship. This finding, while unexpected, points to the qualitatively different effect of HARM in contexts where mentees are not the numerical minority. More specifically, some research supports that judgments of HARM may be seen more negatively when perpetrated by people who do not share the same racial and ethnic background as the mentee based on more negative appraisals of the situations (Woods et al., 2009). Additionally, the qualitative differences in the *type* of HARM beyond the categories of sexual harassment, racial microaggressions, and incivility may look quite *qualitatively* different depending on the lab and mentoring context (e.g., being a numerical minority or not). Future research must attend to these qualitative differences in experiences of HARM among mentees from marginalized backgrounds so as to not gloss over the very real effects that decades of researchers have documented related to these experiences.

### 4.1. Theoretical Implications

This study extends theoretical models of workplace mistreatment in several important ways. Consistent with AET (Weiss & Cropanzano, 1996), the findings confirm that state negative affect functions as a proximal mechanism linking daily mistreatment experiences to program attitudes. Experiences of HARM act as affective events that generate transient emotional responses (e.g., anger, anxiety, frustration, stress), which in turn reduce satisfaction and commitment toward one’s program. This mediation pattern aligns with previous research documenting that negative affect and stress mediate the effects of sexual harassment, incivility, and racial microaggressions on job satisfaction and well-being (Ahmed et al., 2025; Civitillo et al., 2024; Fitzgerald et al., 1997; Lim et al., 2008). By employing a daily design, the present research adds temporal specificity, showing that these emotional consequences occur in real time on a daily basis, not only retrospectively.

Finally, although sexual harassment and racial mistreatment items were too infrequent for separate daily analyses, their inclusion in the HARM composite—and their theoretical positioning alongside incivility—supports the view that diverse forms of interpersonal mistreatment can converge in their proximal affective consequences and downstream attitudinal implications when assessed at the daily level.

### 4.2. Practical Implications

From an applied standpoint, these findings have significant implications for creating academic and organizational environments that align with the call of this special issue to maintain and enrich the lives of individuals at work. First, recognizing that negative affect mediates the effects of HARM underscores the importance of affective regulation and psychosocial safety interventions. Institutions can mitigate these effects through empathy-based training, emotional resilience programs, and mentoring interventions designed to foster empathic leadership and responsive climates.

Second, the moderating role of racial representation reinforces the need for structural and cultural inclusion efforts within laboratories and departments that not only address inclusiveness and psychosocial safety for minoritized members but consider the contextual influence of representation in buffering or exacerbating the effects of HARM/incivility. Ensuring diverse representation at all levels of academic and research environments may reduce both the frequency and impact of HARM experiences. These efforts must go beyond numerical diversity to encompass inclusive climates and equitable mentoring relationships that actively address mistreatment and microaggressions for all trainees (T. D. Allen et al., 2004; Bettencourt et al., 2021).

Third, the daily nature of the effects supports the implementation of real-time monitoring and support systems, such as confidential reporting tools, frequent pulse surveys, check-in mechanisms, and early response systems for addressing mistreatment micro-events before they erode engagement and persistence. Institutions that adopt proactive, responsive measures can foster safer, more inclusive climates that sustain trainee well-being and productivity.

### 4.3. Limitations and Future Directions

Despite these contributions, several limitations warrant acknowledgment. First, the low frequency of severe harassment and racial mistreatment incidents limited the ability to assess sexual and racial harassment independently, necessitating the use of a composite HARM measure where incivility primarily drove effects. Future research with larger, more diverse samples should disentangle these subtypes to identify unique emotional and attitudinal mechanisms. Second, while ESM minimizes recall bias, self-report data may still reflect mood or social desirability effects. Incorporating physiological measures or behavioral indicators could enhance validity. Third, the study focused on biomedical trainees, a high-pressure population characterized by strong hierarchical dynamics; replication across academic disciplines and workplace contexts will test generalizability.

Future research should investigate longer-term outcomes, including whether daily affective responses to HARM accumulate into chronic burnout, withdrawal, or attrition. Moreover, research should assess how organizational climate variables—such as civility norms, inclusion policies, and psychological safety—moderate the emotional and attitudinal pathways identified here. The role of intersectional identities should also be examined to illuminate how overlapping social statuses shape daily emotional reactions and long-term career outcomes.

## 5. Conclusions

This research directly supports the mission of this special issue on the impact of workplace harassment on employee well-being to broaden understanding of workplace harassment and its organizational consequences. By examining multiple forms of mistreatment (sexual, racial, and incivility) simultaneously and identifying state negative affect as a key mediator, this study illustrates how daily interpersonal misconduct can undermine program satisfaction and intended engagement, and persistence. The findings demonstrate that harassment not only harms individuals but also compromises organizational effectiveness, psychological safety, and talent retention—central themes of this issue.

Specifically, we found significant support for the role of affective events theory in the relationship between experiencing HARM and subsequent attitudes about one’s (post)graduate training program. This set of findings expands the current theory of AET to provide evidence of the immediate effects that affective events can have on individual outcomes. Further, we found support that being numerically underrepresented (in addition to socially) in a lab environment or having a mentor who does not share one’s racial identity predicted significantly more experiences of HARM and worse program attitudes. However, contrary to what we expected, we found that in more racially homogeneous labs, the relationship between HARM and program attitudes after accounting for negative affect had an inverse relationship where HARM predicted more favorable program attitudes pointing to a potential buffering effect of being well-represented in a context with others who share similar racial identities. Although we did not expect to find this inverse relationship, this provides a fruitful area for future research to expand to understand the nuanced experiences of HARM in racially monogamous lab settings. Specifically, future research should investigate the role of other more internal factors that might be associated with experiences of HARM on a daily basis among homogenous lab group members other than program attitudes such as physiological and/or psychological safety.

In conclusion, maintaining and enriching work and academic environments requires attention not only to compliance but to daily affective experiences that reveal the human cost of harassment. By documenting the immediate emotional toll of HARM and the protective effects of inclusive climates, this study emphasizes that ensuring healthy, high-functioning environments demands continuous investment in representation, psychological safety, and affective well-being.

## Figures and Tables

**Table 1:** Mentee *N*’s by day.

Variables	Day 1	2	3	4	5	6	7	8	9	10
Sexual harassment	179	175	178	176	170	79	182	170	180	171
Incivility	179	175	178	177	171	80	182	171	182	172
Racial harassment	178	178	178	177	171	80	182	171	182	172
Program Attitudes	174	174	177	177	171	80	182	169	181	170
Negative Affect	174	174	177	177	171	80	182	169	181	170

**Table 2:** Descriptive statistics for the model variables, n = 79–182.

Variable	Average Percent Missing	Possible Score Range	Average Prevalence ^a^	10-Day Prevalence Range	Grand Mean (SD) [10-Day Range]	ICC
HARM		0–28	9.34%	4.95% to 26.50%	0.25 (1.05) [0.10 (0.44) to 0.86 (2.24)	0.30
Program Attitudes		1–5			3.09 (0.60) [3.02 (0.55) to 3.36 (0.77]	0.29
Negative Affect		1–5			1.45 (0.57) [1.36 (0.45) to 1.65 (0.65)]	0.55
Lab gender	1%	1–5			3.55 (1.29)	
Lab race	1%	1–5			2.58 (1.45)	
MMgender	7.4%	0–1			0.41 (0.49)	
MMrace	0.5%	0–1			0.28 (0.45)	

^a^ For HARM, prevalence is the percent of the sample with a score of 1 or higher. For continuous variables, we report the mean and standard deviation. SD = Standard Deviation; ICC = intraclass correlation, HARM = composite of harassment, racial mistreatment, and incivility; MMgender = mentor–mentee gender match (0) = no, (1) = yes; MMRace = mentor–mentee race match (0) = no, (1) = yes.

**Table 3:** Within-level correlation matrix.

	HARM	Program Attitudes
Program attitudes	−0.07	
Negative affect	0.27 ***	−0.34 ***

Note. *** *p* < 0.001. HARM = composite of harassment, racial mistreatment, and incivility.

**Table 4:** Between-level correlation matrix.

Variable	HARM	MMgender	MMrace	Lab Gender	Lab Race	Program Attitude
MMgender	−0.09					
MMrace	−0.55 ***	0.08				
Lab gender	−0.08	0.35 ***	0.23 *			
Lab race	−0.40 ***	0.11	0.48 **	0.14 *		
Program Attitude	0.03	−0.02	−0.09	−0.02	−0.11	
Negative Affect	0.19	0.03	0.09	0.09	−0.06	−0.29 **

Note. MMgender = mentor–mentee gender match (0) = no, (1) = yes; MMRace = mentor–mentee race match (0) = no, (1) = yes. HARM = composite of harassment, racial mistreatment, and incivility. * *p* < 0.05, ** *p* < 0.01, *** *p* < 0.001.

**Table 5:** Moderator analyses of the effect of HARM on program attitudes.

Moderator	B	SE	95% CI	β	*p*
Lab gender	0.02	0.05	[−0.07, 0.12]	0.09	0.61
**Lab race**	**0.08**	**0.02**	**[0.01, 0.16]**	**0.34**	**0.03**
Mm gender	−0.20	0.12	[−0.41, 0.04]	−0.30	0.11
Mm race	−0.07	0.12	[−0.31, 0.15]	−0.09	0.60

Notes: B and β represent the unstandardized and standardized effect of corresponding moderator on the effect of HARM on program attitudes. HARM = composite of harassment, racial mistreatment, and incivility. The significant effects are in bold face, *p* < 0.05.

## Data Availability

An Excel file of the data can be found in the Supplementary Materials.

## Supplementary Materials

The following supporting information can be downloaded at: https://www.mdpi.com/article/10.3390/bs16020214/s1, Supplementary S1: Short Term Effects Data Set with Variable labels. Supplementary S2: Short-Term Effect Supplementary Files.

## Abbreviations

The following abbreviations are used in this manuscript:

HARM	Harassment, Racial Microaggressions, and Incivility
MMRace	Mentor/Mentee Race
MMGender	Mentor/Mentee Gender
