# Supplementary material for: Short-Term Effects of Harassment, Racial Mistreatment, and Incivility (HARM) on Career-Derailing Attitudes: An Experience Sampling Methodology Study
Source: Behav Sci (Basel). 2026 Feb 2;16(2):214. doi: 10.3390/bs16020214 (PMC12938245; doi:10.3390/bs16020214)
Supplement: Supplementary file 1 [file behavsci-16-00214-s001.zip › Supplementary S2. Short-Term Effect Supplementary Files.pdf]

INDIANA UNIVERSITY

APPROVAL LETTER

To: Stockdale, Peggy

Protocol #: 15142

Protocol Title: Biomedical Research mentor/mentee study

Type of Submission: Amendment

Level of Review: Exempt

Approval Date: Wednesday, November 9th 2022

Expiration Date: no date provided

**If Expiration Date = "No date provided," this research does not require annual renewal; thus there is no expiration date.*

The Indiana University HRPP approved the above-referenced submission. Conduct of this study is subject to the IU HRPP Policies, as applicable.

Additional Notes:

Amendment A001

This research is exempt under the following category, and the IRB conducted a limited IRB review:
- Category 2(iii)

Documents approved with this submission:

Attachments

Data Collection Instrument	Mentor enrollment survey.docx
Data Collection Instrument	Mentor Intake survey.docx
Data Collection Instrument	Mentor daily survey.docx
Data Collection Instrument	Mentee intake survey.docx
Data Collection Instrument	Mentee daily survey.docx
Data Collection Instrument	Secondary Mentee survey.docx
Study Information Sheet	Study Information Sheet - Mentors.docx
Study Information Sheet	Study Information Sheet - Mentees.docx
Study Information Sheet	Study Information Sheet - Secondary Mentees.docx
Recruitment Materials	Mentor recruitment messages.docx
Recruitment Materials	Mentee recruitment messages.docx
Recruitment Materials	Secondary Mentee recruitment messages.docx

Data Collection Instrument	Pilot survey_day 1_updated.pdf
Data Collection Instrument	Pilot survey_day 10_updated.pdf
Study Information Sheet	Pilot SIS.docx
Data Collection Instrument	Pilot survey_recruitment survey.pdf
Recruitment Materials	Pilot_Mentee recruitment messages.docx
Study Information Sheet	Video_Intervention_SIS.docx
Recruitment Materials	Video interview phone screener_recruitment messages.docx
Data Collection Instrument	Video_Intervention_Phone Screening Protocol.docx
Data Collection Instrument	Video Interview Protocol.docx
Recruitment Materials	Video interview_recruitment messages.docx

You should retain a copy of this letter and all associated approved study documents in your research records.

If you have any questions or require further information, please contact the HRPP via email at irb@iu.edu or via phone at (317) 274-8289.

Aim 1: Data collection instruments

Instrument 4: Mentee Daily Survey

Instructions: Please complete the following survey items before midnight.

Frequency of Daily interactions

(1) No time at all to (5) All day

1. How much time did you spend with your lab PI <piped text: Professor X>?
2. How much time did you spend with a lab leader, such as a lab supervisor or lab manager today?
3. How much time did you spend with other lab mates today?

Allyship experiences (adapted from Stockdale's adaptation of Ashburn-Nardo et al.'s CPR model (2008)

Scale for all items (1) *not at all*, (2) *somewhat,* (3) *yes, definitely*

1. Someone in my lab supported me in a significant way.
2. Someone in my lab showed that they were an ally toward me.
3. Someone in my lab stepped in to intervene against mistreatment toward me or other lab mates.
4. Someone in my lab mentored me.

Unwanted sexist, sexual, racist, or uncivil experiences

Scale for all items (1) *not at all*, (2) *somewhat,* (3) *yes, definitely*

SEQ (modified from Stark et al., 2002 – SEQ-DoD-short

1. Someone in my lab engaged in sexist behavior toward me or other women.
2. Someone in my lab engaged in sexually crude behavior toward me or other women.
3. Someone in my lab gave me or other women unwanted sexual attention.
4. Someone in my lab implied that I or other women would be treated differently if we cooperated sexually with them.

Incivility (adapted from Cortina et al., 2001)

1. Someone in my lab put me down or was condescending to me.
2. Someone in my lab paid little attention to my opinions.
3. Someone in my lab addressed me in unprofessional terms either publicly or in private

SEQ-L (modified from Cortina, 2001)

1. Someone in my lab engaged in racist behavior toward me or other minorities.
2. Someone in my lab engaged in racially crude behavior toward me or other minorities.

Racial microaggressions (modified from Nadal, 2011 and Torres-Harding et al., 2012)

1. Someone in my lab made assumptions that I or other minorities were inferior.
2. Someone in my lab treated me or other minorities as a second-class citizen.
3. Someone in my lab invalidated me or other minorities experiences as a person of color.
4. Someone in my lab was subtly aggressive toward me or other minorities.
5. Someone in my lab ignored me or other minorities or made us feel invisible.

<If participant selects “somewhat” or “yes, definitely” to any of the **Unwanted sexist, sexual, racist, or uncivil experiences** items above, the following questions will appear.

1. Was the person or persons who did this: (yes/no)
    a. The PI or lead professor of your lab?
    b. Another lab leader, such as a lab manager or senior lab member?
    c. Another lab member
    d. One or more men?
    e. One or more women?
    f. One or more gender nonbinary people?
    g. A combination of men, women, or gender nonbinary people?
2. Coping questions

With regard to the experience(s) you had within the past 24 hours, use the rating scale below to indicate any thing (including doing nothing) you did.

I did nothing   Yes/NO

I told nobody about it Yes/No

I told a friend or family member about it Yes/No

I confronted the person (people) who did this Yes/No

I told a colleague Yes/No

I wrote about it online (e.g. in social media) Yes/No

I sought information and support Yes/No

I made a formal complaint or am making plans to make a formal complaint about it Yes/No

I sought or plan to seek counseling about it Yes/No

I contacted a helpline Yes/No

I contacted Human Resources, Student Affairs, or a Title IX office (or similar) Yes/No

I contacted my union (if applicable) Yes/No

I talked to or plan to talk to a religious/spiritual leader Yes/No

I took some other action Yes/No, If yes provide a text box.

3. Attribution questions (note, we have requested to full set of items from the original authors:

Hershcovis, M. S., & Barling, J. (2010). Comparing victim attributions and outcomes for workplace aggression and sexual harassment. *Journal of Applied Psychology*, *95*(5), 874.

Scale (1: strongly disagree to 5: Strongly agree)

I may have done something to deserve this behavior

I am to blame for my colleague or mentor's behavior toward me

This is personal

My colleague/mentor has it out for me.

This has nothing to do with my gender

This has nothing to do with my race or ethnicity

My colleague/mentor probably behaves this way only towards members of my gender

My colleague/mentor probably behaves this way only towards members of my race

My colleague/mentor is to blame for this

My colleague/mentor is responsible for what happened

My colleague/mentor is at fault for this behavior

2. <text box> If you need assistance with the treatment you experienced today, please contact your institution's Equal Employment office, Title IX coordinator, or someone in Student Affairs. For general assistance contact the National Sexual Assault Hotline -1-800-656-4673. Live chat is available at rainn.org.

Add this question on the Day 10 survey if they indicated experiencing **Unwanted sexist, sexual, racist, or uncivil experiences on any of the daily surveys.**

We would like to follow up with you in about 2 months to complete one more brief survey about your experiences during these past 10 days. Do we have your permission to do so? Yes/No.

Everyone completes these items:

Outcomes such as withdraw intentions, performance, eg.,

Today,

1. My commitment to remaining in this program was:
    a. Much lower than normal
    b. Somewhat lower than normal
    c. About normal
    d. Somewhat higher than normal
    e. Much higher than normal
2. My productivity was:
    a. Much lower than normal
    b. Somewhat lower than normal
    c. About normal
    d. Somewhat higher than normal
    e. Much higher than normal
3. My confidence in my abilities was:
    a. Much lower than normal
    b. Somewhat lower than normal
    c. About normal
    d. Somewhat higher than normal
    e. Much higher than normal
4. My satisfaction in my graduate/post-doc program was:
    a. Much lower than normal
    b. Somewhat lower than normal
    c. About normal
    d. Somewhat higher than normal
    e. Much higher than normal

PANAS – Add the PANAS items after Participants complete the experiences questions. Randomize whether they get the 4 outcome measures listed above vs. The PANAS scale first. Both should come after the experience questions.

This scale consists of a number of words that describe different feelings and emotions. Read each item and then mark the appropriate answer in the space next to that word. Indicate to what extent you have had these feelings in the past 24 hours.

Scale = 1 (not at all); 2 (a little); 3 (Moderately); 4 (Quite a bit); 5 (Very Much)

Enthusiastic

Interested

Determined

Excited

Inspired

Strong

Scared

Afraid

Upset

Distressed

Jittery

Alert

Active

Proud

Attentive

Ashamed

Irritable

Hostile

Guilty

Aim 1: Data collection instruments

Instrument 3: Mentee Intake Survey

<SIS will be embedded here>

I. Demographics

- What is your gender
  - Male
  - Female
  - Gender Non-Binary/Gender-Fluid/Other
- Are you of Hispanic heritage?
  - Yes
  - No
- What is your race/ethnicity (check all that apply)
  - American Indian
  - Asian, Native Hawaiian, Pacific Islander
  - Black
  - LatinX
  - White
  - Other
  - More than one
- Are you an American Citizen?
  - No
  - Yes
- Were you born in the U.S.?
  - No
  - Yes
- What degree are you currently seeking?
  - BA/BS or equivalent
  - MA
  - MS
  - MPH
  - MFA
  - MBA
  - MLS
  - MPH
  - MSW
  - MArch
  - Ph.D.
  - PsyD
  - M.D.
  - MD/PH.D.
  - DSW
  - JD
  - DDS
  - DDM
  - DVM
  - DPH
  - DEng
  - Ed.D
  - Other

- I am in the program pursuing this degree for
  - Years
  - Months
- I have been working in the lab of (or under the mentorship of) <piped text> for
  - Years
  - Months
- In this lab, or under <piped text>’s mentorship there are:
  - One or very few people of the same gender as me
  - Slightly fewer people of the same gender as me
  - About the same number of people with the same gender as me as well as people with a different gender than me
  - Slightly more people of the same gender as me
  - All or almost all of the same gender as me
  - I am the only student in this lab or under <piped text>’s mentorship
- In this lab, or under <piped text>’s mentorship there are:
  - One or very few people of the same race/ethnicity as me
  - Slightly fewer people of the same race/ethnicity as me
  - About the same number of people with the same race/ethnicity as me as well as people with a different race/ethnicity than me
  - Slightly more people of the same race/ethnicity as me
  - All or almost all of the same race/ethnicity as me

II. Stigma Consciousness (from PIetri et al.,

(1) *Strongly Disagree* to (5) *Strongly Agree*

When interpreting “people like me” refer to your race/ethnicity and gender.

- Stereotypes about people like me have not affected me personally (r)
- I never worry that my behaviors will be viewed as stereotypical of people like me (r)
- When interacting with people, I feel they interpret all of my behaviors in terms of my race and/or gender.
- Most people do not judge other people on the basis of their gender and/or race (r)
- Being a person like me does not influence how people act with me (r)

III. Climate for tolerating sexual harassment, racial mistreatment, and incivility

Source (adapted from): Williams, J.H., Fitzgerald, L. F., & Drasgow, F. (1999). The effects of organizational practices on sexual harassment and individual outcomes in the military. *Military Psychology, 11*(3), 303-328

Hulin et al., 1996

Scale for each item (1) not at all; (2) very little; (3) somewhat; (4) a great deal; (5) all the time

1. My mentor/PI enforces policies against
   a. Sexual harassment
   b. Racism, racial harassment, or racial microaggression

    c. Incivility or bullying
2. If someone commits this act, they will be penalized
    a. Sexual harassment
    b. Racism, racial harassment, or racial microaggression
    c. Incivility or bullying
3. If a leader in my lab (PI or other person considered to be a leader) allows this act to occur, they will be penalized
    a. Sexual harassment
    b. Racism, racial harassment, or racial microaggression
    c. Incivility or bullying
4. People in my lab who do this act will likely get away with it (R)
    a. Sexual harassment
    b. Racism, racial harassment, or racial microaggression
    c. Incivility or bullying
5. This act is tolerated in my lab (R)
    a. Sexual harassment
    b. Racism, racial harassment, or racial microaggression
    c. Incivility or bullying
6. Senior leadership in my academic program make an honest and reasonable effort to stop this from happening
    a. Sexual harassment
    b. Racism, racial harassment, or racial microaggression
    c. Incivility or bullying
7. My direct supervisor or mentor makes an honest and reasonable effort to stop this from happening
    a. Sexual harassment
    b. Racism, racial harassment, or racial microaggression
    c. Incivility or bullying
8. Actions are taken at my lab to prevent this from happening
    a. Sexual harassment
    b. Racism, racial harassment, or racial microaggression
    c. Incivility or bullying
9. A person who complains of this in my lab faces risks (R)
    a. Sexual harassment
    b. Racism, racial harassment, or racial microaggression
    c. Incivility or bullying
10. If a person who complains of this in my lab, they will not be taken seriously (R)
    a. Sexual harassment
    b. Racism, racial harassment, or racial microaggression
    c. Incivility or bullying

Don’t use these items:

Read each vignette below and imagine that this has happened in your research lab. Respond to each of the questions that follow.

1. A leader in your lab makes reference to “incompetent women trying to do research tasks they were never intended to do and taking jobs away from better qualified people.” The leader makes all women in the lab feel incompetent and unwanted.

- What is the likelihood of the risk to a woman in your lab if they made a formal complaint?
  - (1) *It would not be any risk* to (5) *It would be extremely risky*
- What is the likelihood this woman’s complaint being taken seriously?
  - (1) *There is a very good chance she would be taken seriously* to (5) There is almost no chance she would be taken seriously.
- What would be done if a woman made a formal complaint?
  - (1) *There would be serious consequences for this leader. The leader would be disciplined* to (5) *Nothing*

2. A leader in your lab talks a great deal about their sex life and tries to get their subordinates to tell them about their personal lives also

- What is the likelihood of the risk to a woman in your lab if they made a formal complaint?
  - (1) *It would not be any risk* to (5) *It would be extremely risky*
- What is the likelihood this woman’s complaint being taken seriously?
  - (1) *There is a very good chance she would be taken seriously* to (5) There is almost no chance she would be taken seriously.
- What would be done if a woman made a formal complaint?
  - (1) *There would be serious consequences for this leader. The leader would be disciplined* to (5) *Nothing*

3. A leader in your lab has said several times that the way for women to get onto good research projects is to “be more friendly and nice” to them.

- What is the likelihood of the risk to a woman in your lab if they made a formal complaint?
  - (1) *It would not be any risk* to (5) *It would be extremely risky*
- What is the likelihood this woman’s complaint being taken seriously?
  - (1) *There is a very good chance she would be taken seriously* to (5) There is almost no chance she would be taken seriously.
- What would be done if a woman made a formal complaint?
  - (1) *There would be serious consequences for this leader. The leader would be disciplined* to (5) *Nothing*

4. One of your lab mates makes frequent remarks about incompetent women doing jobs that they are incapable of doing and refers to them as “affirmative action” hires and “bitches with attitudes” in their presence.

- What is the likelihood of the risk to a woman in your lab if they made a formal complaint?
  - (1) *It would not be any risk* to (5) *It would be extremely risky*
- What is the likelihood this woman’s complaint being taken seriously?

    - (1) *There is a very good chance she would be taken seriously* to (5) There is almost no chance she would be taken seriously.
- What would be done if a woman made a formal complaint?
    - (1) *There would be serious consequences for this lab mate. The leader would be disciplined* to (5) *Nothing*

5. A lab mate continues to pressure the women in the lab to go out with them after they have made it clear that they are not interested.

- What is the likelihood of the risk to a woman in your lab if they made a formal complaint?
    - (1) *It would not be any risk* to (5) *It would be extremely risky*
- What is the likelihood this woman's complaint being taken seriously?
    - (1) *There is a very good chance she would be taken seriously* to (5) There is almost no chance she would be taken seriously.
- What would be done if a woman made a formal complaint?
    - (1) *There would be serious consequences for this lab mate. The leader would be disciplined* to (5) *Nothing*

6. A lab mate has implied that they can make life in the lab very difficult for women by withholding information and interfering with their research unless she has sex with them.

- What is the likelihood of the risk to a woman in your lab if they made a formal complaint?
    - (1) *It would not be any risk* to (5) *It would be extremely risky*
- What is the likelihood this woman's complaint being taken seriously?
    - (1) *There is a very good chance she would be taken seriously* to (5) There is almost no chance she would be taken seriously.
- What would be done if a woman made a formal complaint?
    - (1) *There would be serious consequences for this lab mate. The leader would be disciplined* to (5) *Nothing*

INFORMATION SHEET FOR RESEARCH
Study #15142
Biomedical research mentor/mentee study

About this research
You are being asked to participate in a research study. Scientists do research to answer important questions which might help change or improve the way we do things in the future.

This form will give you information about the study to help you decide whether you want to participate. Please read this form, and ask any questions you have, before agreeing to be in the study.

Taking part in this study is voluntary. You may choose not to take part or may leave the study at any time. Leaving the study will not result in any penalty or loss of benefits to which you are entitled. Your decision whether or not to participate in this study will not affect your current or future relations with Indiana University Purdue University Indianapolis (IUPUI).

Why is this study being done?
The purpose of this study is to understand how PIs' daily moods and thoughts may impact their mentees. This research will inform training and other interventions to increase the effectiveness of mentorship in the biomedical research enterprise in higher education contexts, which in turn should help improve career outcomes for mentees.

You were selected because you are a mentee (graduate student or post-doctoral fellow) of a PI who is funded by NIH. Your PI/mentor's name was culled from NIH's eReporter from which we selected a random sample. Your PI/mentor then gave us your name as a potential participant in this study.
We are inviting 320 PI/mentee pairs to participate in this research. Mentor/PI's survey responses will not be shared with mentees, nor will mentees' survey responses be shared with mentors. We will provide a summary report of our findings at the end of the study.

The study is being conducted <Names and Institution withheld for peer review>. It is funded by NIH/NIGMS.

What will happen during the study?
If you agree to be in the study, you will be asked to complete 11 online surveys (separated by one-week intervals). The first survey (intake survey) will ask you to complete some demographic questions, and questions about your research lab. It will also ask you to complete some personality questionnaires and some questions about the climate of your lab. This survey should take approximately 15 minutes to complete. The next 10 surveys. will ask you about your daily experiences – some are potentially positive, and the others are potentially negative. These surveys should be completed by midnight (your time zone) Monday through Friday across two weeks. The daily surveys should take approximately 10 minutes each to complete. It is

important that you not share your survey responses with your mentors or other lab personnel. You will be prompted to complete each survey with either a text message or an email message. You can complete the daily surveys on a computer or mobile device, such as a smartphone. The daily surveys will be open from 5:00pm your time to 11:59pm your time beginning on the Monday following your completion of the intake survey. We ask that you not skip a survey, but if you do, you will be prompted to complete the next day's survey.

In approximately two months, we will invite some participants to complete another brief online survey. If invited, you may complete this survey any time of the day. It should take about 5-10 minutes to complete. The total amount of time for this study for the 11-12 surveys is approximately 125 minutes.

What are the risks and benefits of taking part in this study?
A risk of completing the surveys is being uncomfortable answering the questions. In this case, please remember that you are free to terminate your participation in this study at any time. There is no penalty for deciding to leave the study.

We don't expect you to receive any benefit from taking part in this study, but we hope to learn things which will help scientists in the future.

How will my information be protected?
All research includes at least a small risk of loss of confidentiality. Efforts will be made to keep your personal information confidential. We cannot guarantee absolute confidentiality. Your personal information may be disclosed if required by law. Your identity will be held in confidence in reports in which the study may be published.

Organizations that may inspect and/or copy your research records for quality assurance and data analysis include groups such as the study investigator and his/her research associates, the Indiana University Institutional Review Board or its designees, and any state or federal agencies who may need to access your research records (as allowed by law).

This research is covered by a Certificate of Confidentiality from the National Institutes of Health. This means that the researchers cannot release or use information, documents, or samples that may identify you in any action or suit unless you say it is okay. They also cannot provide them as evidence unless you have agreed. This protection includes federal, state, or local civil, criminal, administrative, legislative, or other proceedings. An example would be a court subpoena.

There are some important things that you need to know. The Certificate DOES NOT stop reporting that federal, state or local laws require. Some examples are laws that require reporting of child or elder abuse, some communicable diseases, and threats to harm yourself or others. The Certificate CANNOT BE USED to stop a sponsoring United States federal or state government agency from

checking records or evaluating programs. The Certificate DOES NOT stop disclosures required by the federal Food and Drug Administration (FDA). The Certificate also DOES NOT prevent your information from being used for other research if allowed by federal regulations.

Researchers may release information about you when you say it is okay. For example, you may give them permission to release information to insurers, medical providers or any other persons not connected with the research. The Certificate of Confidentiality does not stop you from willingly releasing information about your involvement in this research. It also does not prevent you from having access to your own information.

Will I be paid for participation?
You will receive $150.00 (USD) if you are chosen to participate in the study and if you complete at least 70% of the daily surveys. If you are invited to participate in the two-month follow up survey, you will earn an additional $5.00. Payment will be in the form of an Amazon gift card.

Who should I call with questions or problems?
For questions about the study, contact the researcher, <Contact information withheld for peer review>.

For questions about your rights as a research participant or to discuss problems, complaints or concerns about a research study, or to obtain information, or offer input, please contact the <Institution withheld for peer review>. Human Subjects Office at <contact information withheld for peer review>.

Form Date: September 28, 2023
